# Nanoindentation and XPS Studies of Titanium TNZ Alloy after Electrochemical Polishing in a Magnetic Field

**DOI:** 10.3390/ma8010205

**Published:** 2015-01-08

**Authors:** Tadeusz Hryniewicz, Krzysztof Rokosz, Ryszard Rokicki, Frédéric Prima

**Affiliations:** 1 Division of Surface Electrochemistry, Koszalin University of Technology, Racławicka 15-17, Koszalin 75-620, Poland; E-Mail: rokosz@tu.koszalin.pl; 2 Electrobright, 142 W. Main St., Macungie, PA 18062, USA; E-Mail: ryszardr@ptd.net; 3 Institut de Recherche de Chimie Paris, CNRS–Chimie ParisTech, 11 rue Pierre et Marie Curie, Paris 75005, France; E-Mail: frederic.prima@chimie-paristech.fr

**Keywords:** nanoindentation, XPS studies, magnetoelectropolishing (MEP), TNZ surface

## Abstract

This work presents the nanoindentation and XPS results of a newly-developed biomaterial of titanium TNZ alloy after different surface treatments. The investigations were performed on the samples AR (as received), EP (after a standard electropolishing) and MEP (after magnetoelectropolishing). The electropolishing processes, both EP and MEP, were conducted in the same proprietary electrolyte based on concentrated sulfuric acid. The mechanical properties of the titanium TNZ alloy biomaterial demonstrated an evident dependence on the surface treatment method, with MEP samples revealing extremely different behavior and mechanical properties. The reason for that different behavior appeared to be influenced by the surface film composition, as revealed by XPS study results displayed in this work. The increase of niobium and zirconium in the surface film of the same titanium TNZ alloy after magnetoelectropolishing MEP treatment is meaningful and especially advantageous considering the application of this alloy as a biomaterial.

## 1. Introduction

Titanium and its alloys are advanced metallic materials possessing many interesting features and properties with outstanding corrosion resistance in a wide variety of environments [1,2,3,4,5,6,7,8,9,10,11,12,13]. Because of numerous advantageous properties, they are used in aeronautics, the automotive industry, in jewelry and in biomedical engineering. CP Ti Grade 2 and NiTi alloys have been known for years for their application as biomaterials [1,7,8,9]. Other titanium alloys, such as Ti-6Al-4V, Ti-6Al-4V ELI, Ti-6Al-7Nb, have been used in prosthetic engineering [6,8,9,10], even if they contain carcinogenic vanadium and allergenic aluminum [6,14].

Our earlier studies indicated a considerable dependence of the mechanical properties of titanium samples on the surface treatment method used [2,7,10,11,12,13,14,15,16,17]. It was proven that application of a magnetic field to the process of electropolishing resulted in a significant improvement of the basic mechanical properties [2,13,17]. Some indentation and nanoindentation studies on stainless steels and titanium biomaterials were performed before [2,17,18,19], together with the significance of the quantitative determination of material hardness, stressed in the authors’ other work [15].

The two mechanical properties measured most frequently using indentation and nanoindentation techniques are the hardness *H* or nanohardness *nH* and the elastic modulus *E* [20,21,22,23]. The nanoindentation tests were used by the authors before [2,18] to reveal the effect of magnetoelectropolishing (MEP) in comparison with the results obtained after a standard electropolishing (EP) technology [2,24]. The dependences, relationships and formulae on the depth of indentation, assuming isotropic materials, may be found in the related literature [2,20,25], with the reduced Young’s modulus of the contact between the indenter and the sample *E*_r_ determined by:

(1) $${E_{r}}^{-1}=\frac{1-\nu^{2}}{E}+\frac{1-\nu_{i}^{2}}{E_{i}}$$

where *E* and *v* are the elastic modulus and Poisson ratio for the sample, respectively; and *E_i_* and *v_i_* are the same quantities for the indenter. For the diamond indenter, *E_i_* = 1141 GPa and *v_i_* = 0.07 [25].

The aim of the work is to present the changes in the mechanical and surface film properties of a new, important and advanced titanium biomaterial alloy. Here, TNZ biomaterial is the focus [26]. In the studies, nanoindentation and XPS results obtained on the newly-developed TNZ titanium alloy after some definite heat treatments [26], named AR (as-received), and two finishing electrochemical operations, a standard electropolishing EP and magnetoelectropolishing MEP, are presented.

## 2. Experimental Section

### 2.1. Sample Preparation and SEM Studies

For the nanoindentation studies, new, titanium biomaterial alloy TNZ (Ti 74, Nb 20, Zr 6 wt%) samples were used. Sets of titanium alloys [26], TNZ samples of dimensions of 30 mm × 20 mm × 2 mm, were prepared. Furthermore, the TNZ (Ti-20Nb-6Zr) alloy samples were heat treated at 900 °C.

Both sets of samples were electrochemically treated, the first one under a standard electropolishing (EP) process and the second one by electropolishing in a magnetic field (magnetoelectropolishing, MEP) [2,24,27]. The electrolyte was based on concentrated H_2_SO_4_ with some addition of HF and HNO_3_ acids (80% of 66 Bé° H_2_SO_4_ + 10% of 49% HF + 10% of 71% HNO_3_). The same proprietary electrolyte was used both for EP and MEP processes under the same oxygen evolution regime, above the plateau region. Afterwards, the samples were washed out in a de-ionized water and dried in air. Such prepared samples had undergone nanoindentation studies.

### 2.2. Nanoindentation Testing

Nanoindentation measurements were performed on the 950 TriboIndenter™ Nanomechanical test instrument (Ostrava, Czech Republic) [25]. By using the instrument, quasistatic nanoindentation was applied to measure some mechanical properties, such as Young’s modulus, and nanohardness via nanoindentation. At least twelve imprints were taken each time on every sample investigated.

### 2.3. XPS Studies

The XPS measurements on magnetoelectropolished TNZ alloy samples were performed by means of a SCIENCE SES 2002 instrument (Trondheim, Norway) using a monochromatic (Gammadata-Scienta) Al K(alpha) (*hν* = 1486.6 eV) X-ray source (18.7 mA, 13.02 kV). Scan analyses were carried out with an analysis area of 1 mm × 3 mm and a pass energy of 500 eV with an energy step of 0.2 eV and a step time of 200 ms. The binding energy of the spectrometer has been calibrated by the position of the Fermi level on a clean metallic sample. The power supplies were stable and of high accuracy. The experiments were carried out in an ultra-high-vacuum system with a base pressure of about 6 × 10^−10^ Pa. The XPS spectra were recorded in normal emission. In view of optimizing the signal-to-noise ratio to about 3.2, one XPS measurement cycle covered 10 sweeps. For the XPS analyses, the CasaXPS 2.3.14 software (Shirley background type; Casa Software Ltd., Devon, UK) was used [28,29].

## 3. Results

### 3.1. Nanoindentation Results

**Figure 1:**
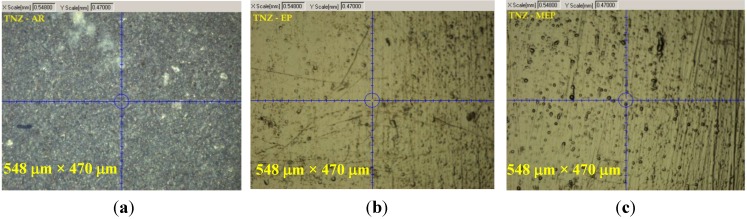
Optical microscope results of TNZ alloy sample surface: AR, as received (**a**); EP, after electropolishing (**b**); MEP, after magnetoelectropolishing (**c**). (window: 548 µm × 470 µm).

**Figure 2:**
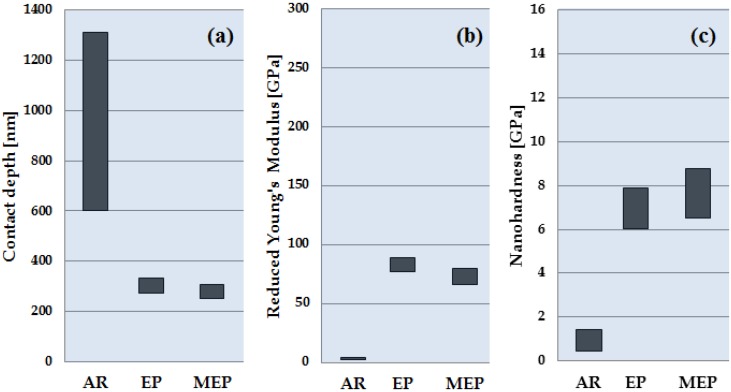
Nanoindentation results of TNZ alloy sample surfaces: (**a**) contact depth; (**b**) reduced Young’s modulus *E_r_*; and (**c**) nanohardness. For the horizontal axis description, see the text.

Figure 1 presents optical microscope results of the TNZ alloy sample surfaces. The presentation of the determination of the reduced Young’s modulus and nanohardness of titanium alloy samples AR and those after EP and MEP treatments are given in Table 1 and in Figure 2. The nanoindentation mechanical data recorded for titanium TNZ alloy samples in three groups, AR, EP and MEP, vary concerning the magnitude and the range of changes. One may notice the differentiation in values of the contact depth, reduced Young’s modulus and nanohardness obtained on the same biomaterial, TNZ, dependent on the state of the material (AR) and the method of the finishing treatment, referring to EP and/or MEP.

**Table 1:** Nanoindentation measurement results of TNZ alloy sample surfaces.

Treatment	Contact Depth (nm)	Reduced Young’s Modulus *E_r_* (GPa)	Nanohardness *nH* (GPa)
AR	957.09 ± 355.21	3.61 ± 0.98	0.94 ± 0.48
EP	303.87 ± 28.79	83.08 ± 5.73	6.97 ± 0.92
MEP	280.67 ± 27.53	72.95 ± 7.21	7.64 ± 1.14

Considering TNZ sample nanoindentation, the contact depth was decreasing from the highest value for the AR sample, well over 950 nm, with the highest variability equaling ±355 nm, down to below 300 nm for the MEP sample and about 13-times less variability, through to EP, which was in between. Thus, the lowest reduced Young’s modulus was found for the AR sample, *E_r_* of about 3.6 GPa, and the variability was below ±1 GPa; however, the highest was for the EP sample, over 80 GPa with a variability of ±6 GPa, with a lower value, 73 GPa, for the MEP sample. The nanohardness results are exactly the reverse of the contact depth value, the lowest *nH* = 0.95 GPa for the AR sample with the variability below ±0.5 GPa and the highest *nH* = 7.64 GPa obtained for the MEP sample with the variability above ±1 GPa, with the EP sample being in between.

### 3.2. XPS Results

**Figure 3:**
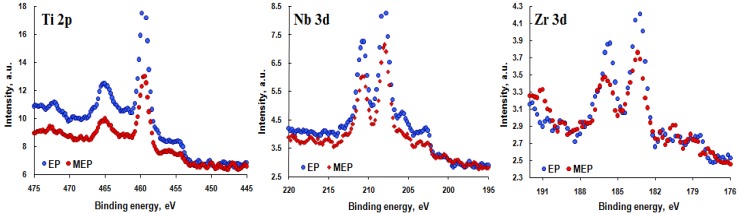
XPS high resolution Ti 2p, Nb 3d and Zr 3d spectra of TNZ alloy sample surface after EP and MEP.

In Figure 3, the titanium **(**Ti 2p), niobium (Nb 3d) and zirconium (Zr 3d) spectra of the passive layer formed after standard EP and MEP are shown. In the presented spectra, the intensities are smaller for MEP than those obtained after EP. The XPS spectra of the titanium 2p_3/2_ and 2p_1/2_ peaks observed at 459.4 eV and 465.2 eV, respectively, can be interpreted as titanium oxide TiO_2_ (Ti^4+^). The spectra of the Nb 3d_5/2_ and Nb 3d_3/2_ peaks at 207.8 eV and 210.6 eV, respectively, can be interpreted as niobium oxide, Nb_2_O_5_ (Nb^5+^) [28]. In the case of zirconium, the Zr 3d_5/2_ and Zr 3d_3/2_ peaks at 183.2 eV and 185.6 eV, respectively, can be interpreted as zirconium oxide, ZrO_2_ (Zr^4+^) [30].

**Figure 4:**
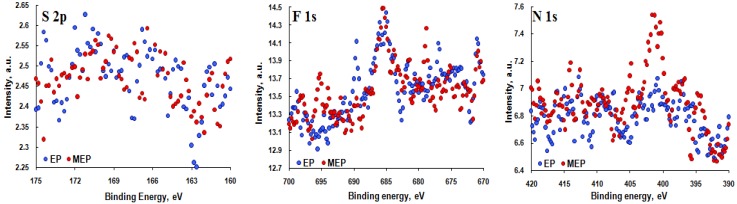
XPS high resolution S 2p, F 1s and N 1s spectra of TNZ alloy sample surfaces after EP and MEP.

In Figure 4, the XPS spectra of elements from the electrolyte, *i.e.* S 2p (*BE* = 166.4 eV and *BE* = 169.4 eV), F 1s (*BE* = 685.4 eV), N 1s (*BE* = 401.2 eV), are presented. Those spectra may indicate the presence of sulfide, sulfates, fluorides and Ti-N-O compounds, respectively [31].

**Figure 5:**
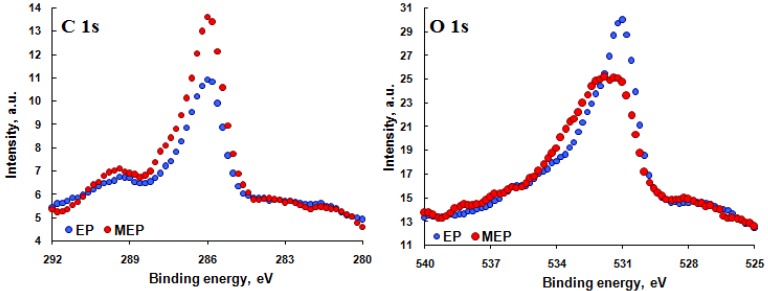
XPS high resolution C 1s and O 1s spectra of TNZ alloy sample surfaces after EP and MEP.

Figure 5 shows the XPS spectra of carbon C 1s and oxygen O 1s. The intensities of these spectra are higher for MEP than those for the EP. This can be explained by the higher contents of titanium-niobium-zirconium with oxygen and/or with sulfur, fluorine and nitrogen compounds, as well as by carbon contaminations.

**Figure 6:**
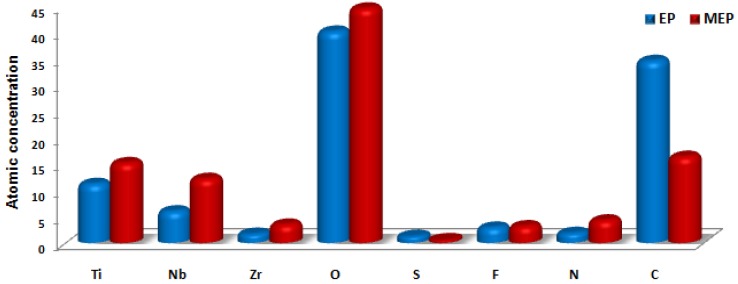
Atomic concentration diagram of the TNZ passive layer alloy after EP and MEP.

In Table 2 and Figure 6, the chemical compositions of the surface layer formed after standard EP and MEP are presented. It is visible that after MEP, in the passive layer, there is about twice more zirconium and niobium and about 1.4-times more titanium than after EP treatment. The high oxygen content detected in the passive layer partly covers the carbon-oxygen contaminations and partly the sulfide, sulfates and Ti-N-O compounds.

**Table 2:** Atomic concentration of detected elements in the passive layer of the TNZ alloy surface after EP and MEP on the basis of high resolution XPS spectra.

Element	EP	MEP
C 1s	35.1	16.4
F 1s	2.9	3.2
N 1s	2.0	4.3
Nb 3d	6.0	12.2
O 1s	39.9	44.6
S 2p	1.3	0.6
Ti 2p	11.1	15.2
Zr 3d	1.7	3.5

In Table 3, the ratios of the main alloy elements to their sum, *i.e.*, Ti/TNZ, Nb/TNZ, Zr/TNZ, are presented. In the TNZ matrix alloy, 74 at% of titanium, 20 at% of niobium and 6 at% of zirconium were registered. After MEP, a smaller amount of titanium (49 at%) than after the EP (59 at%) treatment was recorded. In the case of niobium and zirconium, the contents in the passive layer after EP (32 at%, 9 at%) and after MEP (39 at%, 12 at%), respectively, were detected.

**Table 3:** Calculated ratios of XPS high resolution spectra of TNZ alloy sample surfaces.

Ratio	Matrix	EP	MEP
Ti/(Ti + Nb + Zr)	0.74	0.59	0.49
Nb/(Ti + Nb + Zr)	0.20	0.32	0.39
Zr/(Ti + Nb + Zr)	0.06	0.09	0.12

## 4. Discussion

Two electrochemical processes, EP and MEP, were used for the surface finishing of a newly-developed titanium alloy, TNZ. The magnetic field was used in MEP to additionally modify the sample surface.

In the case of the TNZ (Ti-20Nb-6Zr) alloy, the following electrochemical processes are expected:

$$\text{Ti}+4\text{HF}={\text{TiF}}_{4}+2{\text{H}}_{2}$$

$$\text{Nb}+5\text{HF}={\text{NbF}}_{5}+2{\text{½H}}_{2}$$

$$\left(2\text{Nb}+10\text{HF}=2{\text{NbF}}_{5}+5{\text{H}}_{2}\right)$$

$$\text{Zr}+4\text{HF}={\text{ZrF}}_{4}+2{\text{H}}_{2}$$

with the following reactions of the oxidation of the surface:

$$\text{Ti}+2{\text{H}}_{2}\text{O}={\text{TiO}}_{2}+4{\text{H}}^{+}+4\text{e}$$

$$2\text{Nb}+5{\text{H}}_{2}\text{O}={\text{Nb}}_{2}{\text{O}}_{5}+10{\text{H}}^{+}+10\text{e}$$

$$\text{Zr}+2{\text{H}}_{2}\text{O}={\text{ZrO}}_{2}+4{\text{H}}^{+}+4\text{e}$$

The dissolution of the formed oxides proceeds simultaneously according to the reactions:

$${\text{TiO}}_{2}+6{\text{F}}^{-}+4{\text{H}}^{+}={\left[{\text{TiF}}_{6}\right]}^{2-}+2{\text{H}}_{2}\text{O}$$

$${\text{Nb}}_{2}{\text{O}}_{5}+12{\text{F}}^{-}+10{\text{H}}^{+}=2\;{\left[{\text{NbF}}_{6}\right]}^{-}+5{\text{H}}_{2}\text{O}$$

$${\text{ZrO}}_{2}+6{\text{F}}^{-}+4{\text{H}}^{+}={\left[{\text{ZrF}}_{6}\right]}^{2-}+2{\text{H}}_{2}\text{O}$$

Thus, treated surfaces were scanned to reveal changes in the AR samples after the EP and MEP treatments (see Figure 1 and Figure 2). Afterwards, the basic mechanical properties were studied using a nanoindentation procedure. The nanoindentation measurement results of the titanium alloy biomaterial were obtained for AR, EP and MEP samples, with the results presented in Table 1 and Figure 2. The highest values of the reduced Young’s modulus (*E_r_* = 83.08 ± 5.73 GPa) and nanohardness (*nH* = 6.97 ± 0.92 GPa) were obtained after EP treatment, whereas the lowest values of the reduced Young’s modulus (*E_r_* = 3.61 ± 0.98 GPa) and nanohardness (*nH* = 0.94 ± 0.48 GPa) for the samples without any treatments (AR) were noted. After magnetoelectropolishing (MEP), the medium values of the reduced Young’s modulus (*E_r_* = 72.95 ± 7.21 GPa) and the highest nanohardness (*nH* = 7.64 ± 1.14 GPa) were obtained.

Table 1 covers the mean values and standard deviations of the three studied titanium alloy samples (AR, EP, MEP) [29], which are also presented graphically in Figure 2. One may easily notice the highest deviation value in the nanoindentation contact depth for the AR samples when compared to the values of EP and MEP samples.

In passive layers, formed after electrochemical treatments (EP and MEP) of TNZ, the alloy elements (titanium, niobium, zirconium), elements from the electrolyte (sulfur, fluorine, nitrogen), as well as oxygen from electrochemical process at the transpassive regions of the anodic polarization curve were measured. Additionally, oxygen bound with carbon, in the case of contaminations, was also detected. The analyzes reveal that, most probably in the surface layer, the compounds, such as TiO_2_, Nb_2_O_5_, ZrO_2_, Ti-N-O, as well as sulfide, sulfates, fluorides of titanium-niobium-zirconium, can be detected. After MEP (passive layer composition: 49 at% Ti, 39 at% Nb, 12 at% Zr) in the passive layer of TNZ, less titanium and more niobium and zirconium than after EP (passive layer composition: 59 at% Ti, 32 at% Nb, 9 at% Zr) were measured.

The study results clearly show differentiation in the surface oxide film thickness and composition. A meaningful increase of the expected advantageous elements, niobium and zirconium, in the surface film formed after MEP has been obtained. These findings are of great importance if considering TNZ alloy as a biomaterial with the surface finishing operations. It has been proven that MEP is an interesting means to achieve further modification of the metal surface.

In fact, it should result in the better performance of the titanium TNZ alloy as a biomaterial. That means that one may reduce the cross-section of the material to ensure the same mechanical performance, and *vice versa*. Its biocompatibility is improved by MEP operation. Our findings are in agreement with previous studies performed on CP titanium [2,7,10,11,19] and other metallic biomaterials regarding their resistance to bending [10,13,17,19].

## 5. Conclusions

The Young’s modulus of elasticity and the nanohardness of a newly-developed TNZ titanium alloy were investigated using the nanoindentation technique. The mechanical properties of the TNZ titanium alloy biomaterial demonstrated an evident dependence on the surface treatment method, that is the electropolishing parameters and conditions. After MEP treatment, an improvement in the Young’s modulus of the same Ti alloy biomaterial is observed in comparison with the results obtained after a standard EP.

Moreover, the XPS results have shown that there is a difference in the surface layer chemical composition formed after standard EP and MEP. After both of these electrochemical treatments (EP, MEP), the same elements were detected, which were mentioned in the Discussion; however, the ratios of these elements were substantially changed. The least content of titanium and the highest for niobium and zirconium after MEP and the highest content of titanium and the least for niobium and zirconium on TNZ alloy without any treatment (AR) were registered.
